# The power of genomic estimated breeding values for selection when using a finite population size in genetic improvement of tetraploid potato

**DOI:** 10.1093/g3journal/jkab362

**Published:** 2021-10-21

**Authors:** Catja Selga, Fredrik Reslow, Paulino Pérez-Rodríguez, Rodomiro Ortiz

**Affiliations:** 1 Department of Plant Breeding, Swedish University of Agricultural Sciences (SLU), Lomma SE-23422, Sweden; 2 Colegio de Postgraduados (COLPOS), CP 56230, Montecillos, Edo. de México, Mexico

**Keywords:** genomic selection, potato breeding, tuber yield, tuber quality

## Abstract

Potato breeding relies heavily on visual phenotypic scoring for clonal selection. Obtaining robust phenotypic data can be labor intensive and expensive, especially in the early cycles of a potato breeding program where the number of genotypes is very large. We have investigated the power of genomic estimated breeding values (GEBVs) for selection from a limited population size in potato breeding. We collected genotypic data from 669 tetraploid potato clones from all cycles of a potato breeding program, as well as phenotypic data for eight important breeding traits. The genotypes were partitioned into a training and a test population distinguished by cycle of selection in the breeding program. GEBVs for seven traits were predicted for individuals from the first stage of the breeding program (T_1_) which had not undergone any selection, or individuals selected at least once in the field (T_2_). An additional approach in which GEBVs were predicted within and across full-sib families from unselected material (T_1_) was tested for four breeding traits. GEBVs were obtained by using a Bayesian Ridge Regression model estimating single marker effects and phenotypic data from individuals at later stages of selection of the breeding program. Our results suggest that, for most traits included in this study, information from individuals from later stages of selection cannot be utilized to make selections based on GEBVs in earlier clonal generations. Predictions of GEBVs across full-sib families yielded similarly low prediction accuracies as across generations. The most promising approach for selection using GEBVs was found to be making predictions within full-sib families.

## Introduction

Potato (*Solanum tuberosum* L.) has become one of the most popular food crops worldwide since its introduction from South America to Europe in the 16th century, and thereafter its broad spread to other regions (Bradshaw and Ramsey 2009). Potato is a major source of carbohydrates in human nutrition, and a main component of several industrial applications. Efforts to enhance potato tuber traits have been ongoing since cultivation first began, over 12,000 years ago between south-eastern Peru and western Bolivia (Spooner *et al.* 2005).

Modern potato breeding was initiated in 19th century in Europe (Knight 1807). Most modern potato cultivars are polyploid with tetrasomic inheritance (2*n* = 4*x* = 48). Due to random chromatid segregation and double reduction (a phenomenon which depends on the specific position of a gene on a chromosome), it can be very hard to predict genetic segregation in the offspring derived from a cross between two tetraploid parents (Spooner *et al.* 2014). The potato genome consists of 12 chromosomes and has a haploid size of approximately 840 Mb (Potato Genome Sequencing Consortium 2011). Since the sequencing of the potato genome, there has been an increase in genetic research on both tetraploids and related diploids.

Gene mapping using linkage analysis or association genetics is more successful for, and therefore often limited to, traits with high heritability. Some traits have been successfully mapped using a genome wide association study approach or linkage analysis in a derived bi-parental population, *e.g.*, host plant resistance to late blight (LB), nematodes and viruses, glycoalkaloid and starch content, flesh color, and tuber traits including yield (Ramakrishnan *et al.* 2015, and references therein). Reliable genetic mapping of tuber yield traits is a challenging research task due to low to medium narrow-sense heritability (0.142–0.291; Ortiz and Golmirzaie 2002) and the large number of quantitative trait loci (QTL) with very minor effects (Schönhals *et al.* 2017).

Early DNA marker research on potatoes was conducted using restriction fragment length polymorphisms (RFLPs), amplified-fragment length polymorphisms (AFLPs), and simple sequence repeats (SSRs), and a small number of single nucleotide polymorphisms (SNPs). However, since the development of the first high-density SNP array in 2011, which included 8303 SNPs (Hamilton *et al.* 2011), the accessibility of genomic information for potato has increased. The latest such high-density SNP array, which was developed by Vos *et al.* (2015), contains more than 22,000 SNPs. As the number of genotyping methods increase and the technology develops, the costs of genotyping large populations have decreased, and this has increased the availability of high-throughput genotypic data for small-to-medium size breeding programs.

Genomic prediction is a technique that promises more precise selection and short generation intervals for the improvement of breeding efficiency. The technique has been successfully implemented in dairy cattle, pig and poultry breeding, and more recently in plant breeding, particularly for cereals, though it remains in its infancy for many crops including potato (Ortiz 2015). Genetic gains in potato breeding remain small vis-à-vis other main crops that feed the world (Gao 2021). For example, Douches *et al.* (1996) did not find any progress trend for tuber yield in North American cultivars released during the 20th century, although they showed improvements in quality traits for various end-users according to skin color type and the fresh and processing markets. GEBV for selection in potato breeding may be one approach to overcome this limitation (Slater *et al.* 2016), particularly when costs of high throughput genotyping with dense DNA markers are falling and the number of such markers does not need to be high either for genomic prediction or for association genetics (Selga *et al.* 2021). The major advantage of genomic selection compared to conventional, phenotype-based selection is the possibility of selecting individuals before they are planted in the field. This is done by recording phenotypic and genotypic data for a set of individuals, referred to as a training population, then using this information to fit statistical models that allow the prediction of the performance of a new set of related individuals, often referred to as a test population or breeding population, for which only genotypic information is available (Desta and Ortiz 2014, and references therein). Genomic selection requires high density markers across the whole genome and depends on the assumption that at least one genetic marker will be in linkage disequilibrium with a causative QTL for the trait of interest (Meuwissen *et al.* 2001). The success of genomic prediction models depends not only on the heritability of the trait of interest, but also on the relationships of the individuals included in the training and test populations (Habier *et al.* 2007). The performance of genomic prediction models is determined by the predictive ability of the model to determine the breeding values of the individuals in the test population based on the information provided. Cross-validations of genomic prediction models are often performed; in these, a set fraction of a population with genotypic and phenotypic data is used to predict the behavior of a smaller fraction of the population where the phenotypic information has been masked, taking a new fraction of the population for each round of prediction. The fraction of the population used as a test population (where the phenotypic information has been masked) is often set at around 20% (José Crossa, CIMMYT, pers. comm.). However, in real-life scenarios in breeding programs, the method of repeatedly cross-validating the prediction models does not reflect the conditions of predictions made over separate generations of selection. The cross-validation approach may also overestimate the accuracy of prediction as the relationship between the individuals becomes very strong when the set of individuals used in the training population changes with each run of the prediction model (Windhausen *et al.* 2012). Plant breeders would like to identify as potential parents those whose genomic estimated breeding values (GEBVs) are separated from those of the original training population in both time and space. Hence, using the same unique set of individuals consistently in training and test populations respectively may yield more realistic prediction accuracy than repeated cross-validation.

In recent years, a few research articles about estimating GEBVs for important breeding traits in potato such as, among other characteristics, tuber dry matter content (or specific gravity [SG]), tuber yield, number of tubers per plant, potato chipping quality and starch content in the tubers, have become available. Some of them estimated prediction accuracy using several fold cross-validation (Habyarimana *et al.* 2017; Enciso-Rodriguez *et al.* 2018; Stich and Van Inghelandt 2018), while others validated their models using distinct training and test populations (Sverrisdóttir *et al.* 2017, 2018; Endelman *et al.* 2018). Endelman *et al.* (2018) used data from 11 public potato breeding programs in the USA and the recorded clones and cultivars as a training population for genomic prediction applied to unselected potato clones, often referred to as T_1_s in a potato breeding program, for traits such as SG and tuber yield.

The objective of this research was to assess the potential of genomic selection for eight breeding traits, including tuber yield and quality, using varied genetic backgrounds of available germplasm in a finite size potato breeding program in Sweden, which, as happens elsewhere when funding for public breeding is limited, may be regarded as small or having below 10,000 T_1_ every year going to the field for testing (Eriksson *et al.* 2016). Our proposed method suggests that the more advanced breeding material, *i.e.*, all individuals that underwent at least one cycle of selection (often referred to as T_2_, T_3_ up to T_n_ where *n* is the number of advanced generations), should be used as a training population for the unselected material in T_1_. We also test the approach of predicting phenotypic data across full-sib families of unselected material in T_1_, to ascertain whether it is possible to reduce the number of families that needs to be scored in early clonal generations.

## Materials and methods

### Plant material

The experiments included 669 tetraploid potato clones, which were selected from three distinct populations at different stages of selection (hereafter denoted T_1_, T_2_, and T_3__–__7_) from the potato breeding program managed by the Swedish University of Agricultural Sciences (SLU) in Alnarp, Sweden. The breeding populations were bi-parental offspring derived after crossing cultivars of tetraploid table potatoes, or elite breeding clones from this breeding program (Table  1 and Supplementary Tables S1 and S2). Population T_1_ was limited to five bi-parental families (denoted T_1_–A, T_1_–B, T_1_–C, T_1_–D, and T_1_–E) that had not undergone any previous selection. Four of the parents or grandparents for these families were also included in our research—“Bionica,” “Desiree,” “Sarpo Mira,” and “SW93-1015” (Table  2). The numbers of individuals differed between populations and full-sib families (Tables  1 and 2). The T_1_ population was larger than the other two, more advanced, breeding populations, but this mirrors the real conditions in a commercial potato breeding program, where each year the number of genotypes is reduced while the numbers of observations per genotypes are increased (Bradshaw 2017). The individuals in population T_2_ had been selected once in field trials. Population T_3__–__7_ contains individuals that had been selected during multiple consecutive years of field trials, ranging from 2 to 7 years.

**Table 1:** Size, number of crosses used to produce the individuals and selection cycles for each of the three populations included in this study, T_1_, T_2_, and T_3–7_

Population	T_1_	T_2_	T_3–7_
Size (number of individuals)	465	138	62
Number of crosses	5	29	33
Cycles of selection*	0	1	2–8

**Table 2:** Full-sib families of population T_1_ with information on the number of individual genotypes for each parental cross, as well as the parents of the five crosses

Family	Size (number of individuals)	Crossing parents
A	51	“Bionica” × “Sarpo Mira”
B	51	“C08II69”^**^ × “Bionica”
C	149	“L17”^*^ × “Sarpo Mira”
D	152	“L26”^*^ × “Sarpo Mira”
E	62	“L4”^*^ × “Sarpo Mira”

* “L4,” “L17,” and “L26” are full-sibs from a bi-parental cross between breeding clone “SW93-1015” and cultivar “Desirée.” Data from these two “grandparents” were also recorded alongside the data from T_1_.

** “C08II69” has “SW93-1015” as one of its parents.

### Genotypic data

Leaf material, approximately 0.25 g, was collected on ice from each of the 669 individuals and five cultivars as checks. The leaf material was then sent to SGS—TraitGenetics GmbH for DNA extraction, genotyping, and allele scoring. Genotyping was conducted using the GGPv3.0 array (Vos *et al.* 2015) containing circa 22K SNPs. Quality control for the extracted DNA was conducted by running samples on a gel to check for fractionation. All samples were found to be suitable for further analysis. Genotype calling was done using the software “Illumina GenomeStudio” (Illumina, San Diego, CA, USA), with four alleles scored per locus. After removing SNP markers with more than 10% missing scores, 14884 markers remained. Missing values were imputed with Population mode which deploys the most frequent allelic state using the R package “GWASpoly” (Rosyara *et al.* 2016). To ensure a genotype matrix with polymorphic markers, those SNP markers with a minor allele frequency smaller than 0.05 were discarded. The number of markers available for analysis following marker filtration based on minor allele frequency differed depending on the individuals included. When all individuals were included (*n* = 669), the number of markers was 10,546. For the smaller sets, including 647 and 200 individuals, the total numbers of markers after filtration were 10,499 and 11,219, respectively. The allelic states were translated from base pairs to numeric format to facilitate further processing of genomic data. The numeric data consisted of five allelic states, ranging from 0 to 4, in which 0 and 4 represent the two homozygotes OOOO and AAAA, and 1, 2, and 3 represent the three possible heterozygotes AOOO, AAOO, and AAAO, respectively.

Similarity between individuals was used as an additional quality control measure for the SNP array. Allele callings were compared between individuals over all markers. Individuals genotyped twice were compared to each other, with an expectation of very small differences between allele callings. Related and unrelated individuals were compared to each other, with a higher degree of difference expected. Similarity checks of genotypic data were done using the R statistical package (R Core Team 2021).

To reveal whether there was any population structure among the individuals, a Euclidian distance matrix was calculated based on marker data. Using the R package “adegenet,” a principal coordinate analysis (PCoA) was conducted with the Euclidean distance matrix (Jombart 2008; Jombart and Ahmed 2011). The PCoA was visualized using the R package “ggfortify” (Tang *et al.* 2016; Horikoshi and Tang 2018).

### Phenotypic data

The tubers for the field experiments were brought up from plants grown in a greenhouse for population T_1_, and from field grown plants in northern Sweden (84°N, 20°26′E) for T_2_ and T_3__–__7_, to limit the number of virus-infected tubers. Data from the three populations were recorded at three sites in Sweden (Helgegården, Mosslunda—both in Skane in south Sweden—and Umeå in the north) in 2018. For population T_1_, data were taken at Mosslunda (55°98′N, 14°10′E), with one repetition of, in total, four observations in 2018. Host plant resistance to foliar LB, caused by the pathogen *Phytophthora infestans*, was scored during the cropping season in 2016, from 21st July (first sighting of the disease in the field) until 29th September. Data from T_2_ were recorded in the same field as the T_1_ in 2018; *i.e.*, from 10-plant plots. The data from T_3__–__7_ were collected at the same field site as T_1_ and T_2_ and for 38 of its breeding clones at two additional sites (Helgegården: 56°02′N 14°07′E and Umeå: 63°84′N 20°26′E) in 2018 using 20-plant plots. The tubers of all T_i_s were sown between 14th and 15th May, and they were harvested on 11th and 12th October. Five cultivars (“Bintje,” “Carolus,” “Connect,” “King Edward,” and “Solist”) were planted at all three field sites. All phenotypic records were adjusted by location using the means of the five cultivars from each location, with the exception of LB which was scored only on a per-plant basis.

Phenotypic data for eight important breeding traits were recorded in the field during the cropping season and after harvest. For each plant, tuber weight (TW) was measured in kilograms and tuber number (TN) was recorded at harvest. The average tuber weight (ATW) measured in kilograms was calculated by dividing TW by TN. Likewise, at harvest, tuber uniformity, Eye depth, Shape, and Size (from now on referred to as Eye, Shape, and Size) were estimated using a discrete scale ranging from 1 (nonuniform) to 9 (uniform). Finally, SG was recorded as the weight of tubers in air divided by the weight of tubers in water. TW, TN, and ATW were recorded for all three populations (648–669 genotypes) across all sites (with one or two replicates per site). Eye, shape, size, and SG were recorded for populations T_2_ and T_3__–__7_ (200 genotypes) across all sites (with one or two replicates per site). LB was scored only in the T_1_ population (465 genotypes), and data were collected six times over the course of plant growth. These scores were used to estimate the area under disease progress curve (AUDPC) according to known international standards (Forbes *et al.* 2014). Analysis of variance (ANOVA) was conducted for each trait in order to study differences in performance among the populations. For the traits collected for T_1_, Tukey’s range test of means (Tukey 1949) was conducted to determine any differences among the five families.

### Heritability estimates

Trait heritability was estimated using 38 clones present at all three locations. Broad-sense heritability (*H*^2^) was estimated for all traits by calculating the genetic variance components for genotype using the following equation:

(1) $$H^{2}=\frac{\sigma_{g}^{2}}{\sigma_{g}^{2}+\left(\frac{\sigma_{g\times e}^{2}}{Hm1}\right)+\left(\frac{\sigma_{E}^{2}}{Hm2}\right)}$$

where $\sigma_{g}^{2}$ is the variance of the genotype, $\sigma_{g\times e}^{2}$ the variance due to genotype and environment interaction and $\sigma_{E}^{2}$ the residual variance (Cullis *et al.* 2006). *Hm1* is the harmonic mean of repetitions of each genotype at each location, and *Hm2* is the harmonic mean of the number of repetitions of each genotype across all locations. The variance components $\sigma_{g}^{2}$, $\sigma_{g\times e}^{2}$, and $\sigma_{E}^{2}$ were obtained using the package ASReml-R version 3.0 (Butler *et al.* 2018), after fitting a linear mixed model that includes genotype, environment, genotype by environment and replicate per location as random terms. The R statistical package was used for the statistical analysis (R Core Team 2021).

### Genome-wide predictions of phenotypic traits

The R package “BGLR” (Pérez and de los Campos 2014) was used to fit the prediction models and estimate breeding values (GEBVs) for the phenotypic traits. To estimate GEBVs, the Bayesian Ridge Regression model (equivalent to the commonly used GBLUP model), was fitted. The model is:

(2) y=1μ+Xβ+e,

where ***y*** is the response vector, μ is an intercept, X is the matrix of markers, $x_{ij}\in \{0,1,2,3,4\}$, which corresponds to the allelic dosage, ***β*** is a vector of marker effects and e is the vector of residuals assumed to be distributed normally with mean 0 and variance covariance matrix $\sigma_{e}^{2}I$, with $\sigma_{e}^{2}$ a variance component associated to the residuals. We assigned a multivariate normal distribution with mean **0** and variance $\sigma_{\beta }^{2}I$ to β, with $\sigma_{\beta }^{2}$ a variance component associated to the markers, so that all marker effects have the same shrinkage (Gianola 2013).

### Cross-population analysis

We partitioned the data into two sets, training and test, in order to study the model’s predictive ability. The sets were assigned based on generations: T_1_, T_2_, T_3__–__7_ and the combination of T_2_ and T_3__–__7_ (hereafter referred to as T_2__–__7_). To increase the genetic overlap between populations, the parents and grandparents of the full-sib families in T_1_ were included in the population T_2__–__7_ for prediction of the tuber yield traits. The set with individuals at later stages of selection was assigned as the training set, which was used to train the model to generate GEBVs for the test set made up of individuals from earlier generations in the breeding program. For each partition, we fitted model (2) and we computed the Pearson’s correlation coefficient between observed and predicted values. The model was fitted using the Bayesian framework with the BGLR package (Pérez and de los Campos 2014). Inferences were based on 30,000 samples after discarding 15,000 samples that were used as burn-in.

### Cross-family analysis

Cross-family analysis was conducted for two of the full-sib families from the first clonal generation (T_1_-C and T_1_-D). The families were assigned to the training or test set separated by family, and the model's predictive ability was assessed as described for cross-population analysis in the section above.

### Cross-validation analysis

The population was also randomly divided using fivefold schemes for each of the seven traits. The individuals were randomly assigned to five disjoint groups, where one acted as test set and the other groups as training set, and the process was repeated until all individuals had been given a predicted phenotypic value. For each partitioning we fitted model (2) following the same procedures as described in the previous section. Each fivefold validation was run 100 times, with new randomly assigned groups. The mean prediction accuracy for the cross-validation was calculated as the mean Pearson’s correlation coefficient between observed and predicted values. Cross-validation was also conducted within two full-sib families from the first clonal generation (T_1_-C and T_1_-D). The partitioning and model fitting followed the same approach as when all individuals were included.

## Results

### Genotypes and population structure

Genotyping of 669 individuals from three breeding populations from various stages of a potato breeding program (including the parents and grandparents of some of these offspring) was done using an SNP array. There were 10,546 polymorphic SNPs of high quality for all the individuals of the breeding populations. The SNP array was deemed robust, as less than a 0.5% difference in genotype calling was found among samples collected from the same individual, while on average randomly drawn individuals from the data set had a difference of 50% between the genotype callings.

**Figure 1:**
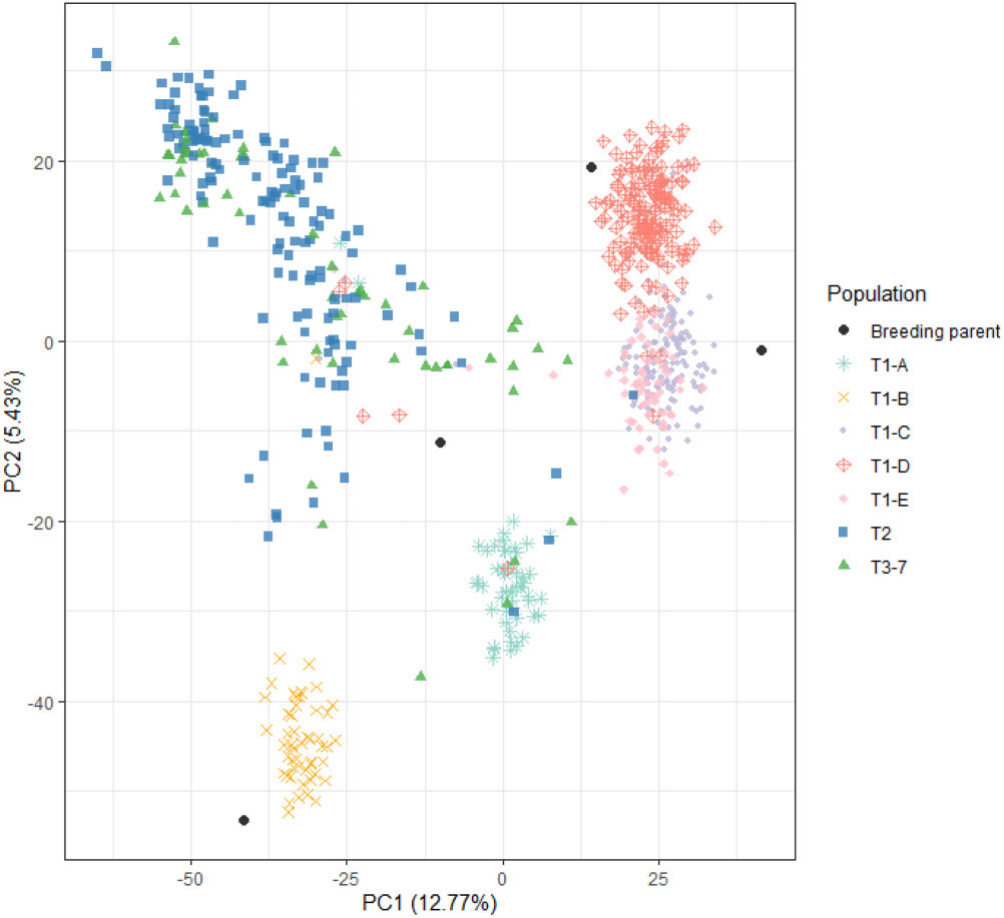
Principal coordinate analysis (PCoA) based on the Euclidian genetic distance between genotyped individuals. Colored groupings are made based on breeding populations across various selection cycles, T_1_–T_3–7_. The five full-sib families of population T_1_ are also separated by color. Four cultivars or breeding clones from the bi-parental crosses that produced the T_1_ families are included as breeding parents. PC1 and PC2 are the % variance explained by the two first principal components.

A PCoA including the polymorphic markers of the 669 individuals revealed clear population structure (Figure  1). The first principal coordinate (PC1) explained 12.77% of the variation in the genotypic data set while the second principal component (PC2) accounted for 5.43% of this variation. There was distinct separation of the more elite breeding material (T_2_ and T_3__–__7_), which included more bi-parental families than the T_1_ (consisting of only five bi-parental families). No such strong population structure due to genotypic data was found among the later stages of selection. The population structure was also studied for population T_1_ (Supplementary Figure S1), and T_2_ and T_3__–__7_ (Supplementary Figure S2) by conducting separate PCoAs based on the Euclidean distance matrix computed using the markers for the individuals within each of the two populations. These plots revealed the same results as the PCoA containing data from all individuals, with populations T_2_ and T_3__–__7_ having less defined population structure than the five families from population T_1_. The % variance explained by the two first PCs was slightly higher for the T_1_ population (PC1 accounted for 11.87% and PC2 accounted for 7.24%) than for the T_2_ and T_3__–__7_ populations (PC1 accounted for 9.45% and PC2 accounted for 5.98%). The structure among the five T_1_ full-sib families was distinct, with a majority of the full-sibs clustering together (Supplementary Figure S1). T_1_-C, T_1_-D, and T_1_-E, which all share a male crossing parent and where the female crossing parent are full-sibs, seem to be at a closer genetic distance compared to the other two families. T_1_-A share one crossing parent with T_1_-B and one with T_1_-C, T_1_-D, and T_1_-E. The individuals included in T_1_-A were at an equal distance to T_1_-B and the other three half-sib families.

### Phenotypes and heritability

**Figure 2:**
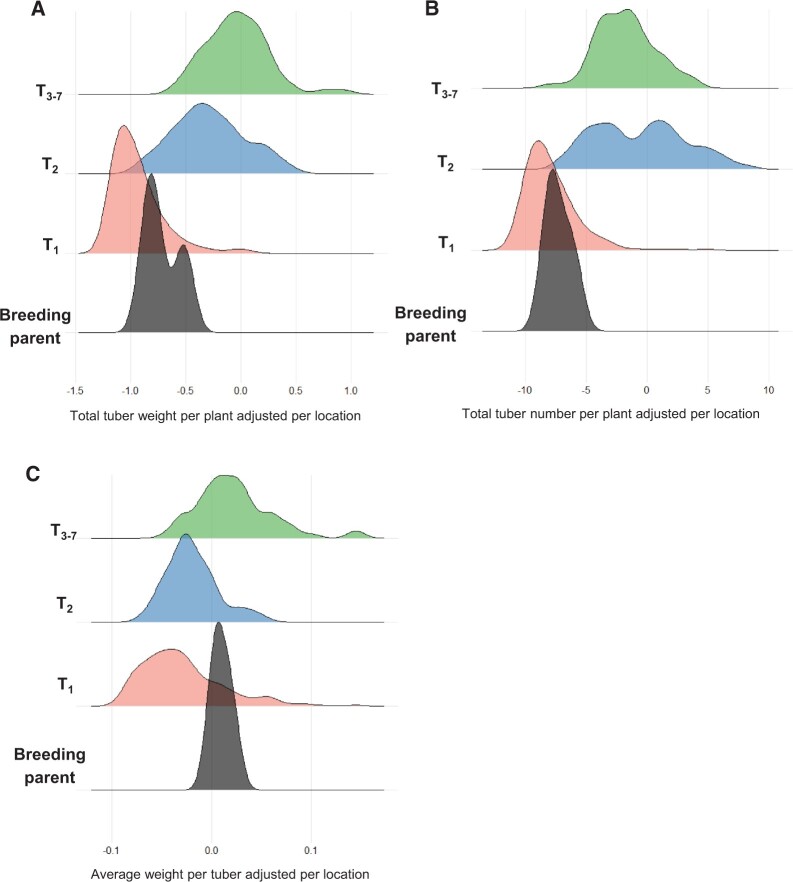
Phenotypic scores for (A) TW per plant, (B) TN per plant, and (C) average TW, across breeding cycles in the potato breeding population and clones and cultivars representing the breeding parents, *y*-axis. Phenotypes are adjusted per location by the means of five cultivars used as checks.

**Figure 3:**
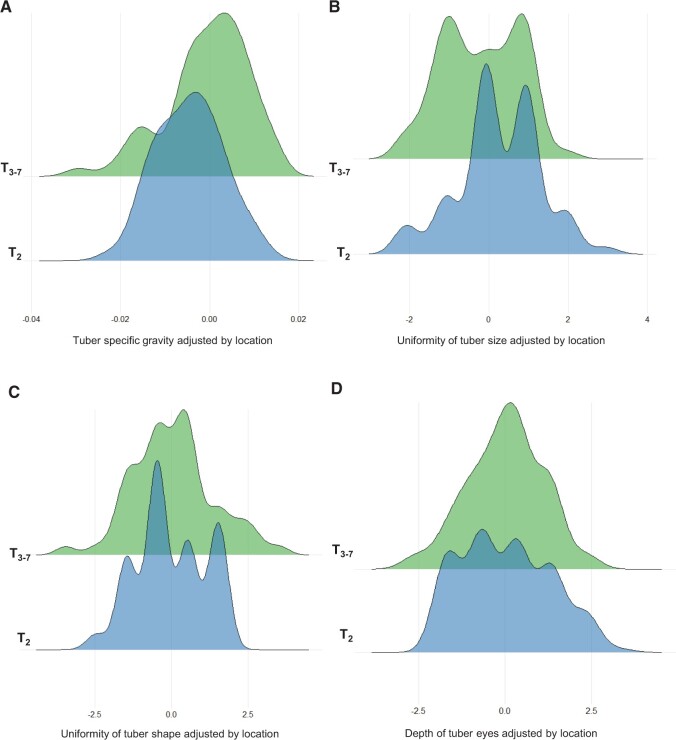
Phenotypic scores for (A) SG, (B) uniformity of tuber size, (C) uniformity of tuber shape, and (D) tuber eye depth, across breeding cycles in the potato breeding population, *y*-axis. Phenotypes are adjusted per location by the means of five cultivars used as checks.

As the data were taken from several field sites, the means adjusted by site were calculated to allow comparisons within and amongst the populations. There were differences in performance for the tuber yield traits (TW, TN, and ATW) among the three populations T_1_, T_2_, and T_3__–__7_ (Figure  2, A–C), and these differences were found to be significant (Supplementary Table S3). T_1_ had on average much lower scores for TW and TN than T_2_ and T_3__–__7_ (*P < *0.001) (Figure  2, A and B). For ATW, the individuals in T_2_ were more similar to the individuals in T_1_, while individuals from T_3_ had higher scores (Figure  2C). The parents or grandparents of the five T_1_ families showed a poor performance for TW and TN, but slightly higher than the mean of the T_1_ population (Figure  2, A and B). However, for ATW, their performance was similar to that of T_3__–__7_ individuals (Figure  2C). ANOVAs were conducted to determine the difference between the two populations separated by time (T_2_ and T_3__–__7_). There were significant differences in SG and Size between the T_2_ and T_3__–__7_ populations (Figure  3, A and B; Supplementary Table S3). The populations did not differ for Shape and Eye (Figure  3, C and D, Supplementary Table S3). Data for four phenotypic traits were available for the T_1_ population (TW, TN, ATW, and LB). The phenotypic scores among the five full-sib families in T_1_ were very similar, but some significant differences were detected (Supplementary Figure S3, A–D, Table S4). Families T_1_-C and T_1_-D were the two largest among the five families, and were studied in more detail in subsequent analysis. Between these two families, TW, TN, and ATW differed (*P < *0.001), while no difference was found for LB.

The *H*^2^ values for the three tuber yield traits—TW, TN, and ATW—were moderately high at 0.64, 0.668, and, 0.692 respectively (Table  3). Both Eye and SG had high *H*^2^ estimates, 0.622 and 0.702. On the other hand, Size and Shape had low heritability: 0.094 and 0.016, respectively (Table  3). Trait heritability, based on variance components, was estimated for the whole population, and for the same individuals as *H*^2^, separately (Table  3). Phenotypic variation estimates will depend on the trait scoring system used, and therefore comparisons between traits were not pursued.

**Table 3:** Broad-sense heritability and variation for the seven phenotypic traits: tuber weight per plant (TW), tuber number per plant (TN), average weight per tuber (ATW), SG and uniformity of tuber size and shape and tuber eye depth

	TW	TN	ATW	SG	Size	Shape	Eye
Heritability	0.64	0.668	0.692	0.702	0.094	0.016	0.622
Variation	0.125	9.308	0.002	2.9 × 10^−4^	2.285	4.902	1.469
Total variation	0.815	16.11	0.002	6.8 × 10^−5^	1.067	1.414	1.488

Heritability and variation were calculated for the 38 individuals present at all three locations. The total variation represents the phenotypic variation among all individuals scored (*n* = 200–669).

### Evaluation of predictive power across populations

GEBVs were predicted for each of the seven phenotypic traits using the Ridge Regression to estimate individual marker effects. To test the effectiveness of predicting traits across populations, the phenotypic values of the population with individuals from the two first stages of the breeding program (T_1_ or T_2_ depending on trait data availability) were removed. By building a prediction model utilizing the phenotypic data from individuals at later cycles of selection in the breeding program as well as the genetic relationship between individuals, the phenotypic values could be estimated. A phenotypic trait was said to be predictable if the prediction accuracy was above 0. The linear model was able to predict all traits. The predictive ability varied greatly across phenotypic traits.

There was no general trend as to a trait’s heritability and the predictive ability of GEBVs. The tuber yield traits—TW, TN, and ATW—were predicted using two different assignments of training and test sets, as these were the only traits that were collected for population T_1_. The model’s predictive ability did not differ depending on which populations were included, except for ATW (Table  4). The prediction accuracy was increased from 0.04 to 0.18 when population T_1_ was excluded, allowing T_3__–__7_ to act as a training set to predict phenotypic values for T_2_. Overall, the lowest prediction accuracy was for TW, TN, and Shape (Table  4). SG had the highest prediction accuracy, *i.e.*, 0.43. Size and Eye also showed moderately high predictive ability.

**Table 4:** Prediction accuracy (correlation between observed and predicted phenotypes) for seven breeding traits: tuber weight per plant (TW), tuber number per plant (TN), average weight per tuber (ATW), SG and uniformity of tuber size and shape and tuber eye depth

Training population	Test population	Breeding trait						
		TW	TN	ATW	SG	Size	Shape	Eye
T_2−7_	T_1_	0.05	0.05	0.04				
T_3−7_	T_2_	0.07	0.04	0.18	0.43	0.16	0.03	0.15
80%^*^	20%^*^	0.75	0.72	0.39	0.62	0.17	0.045	0.15

Model validation was conducted either on the populations divided by cycle of selection, or by randomly partitioned fivefold cross validations.

* For the cross validation the total population was divided at random, mean prediction accuracies over 100 model runs.

### Evaluation of predictive power of cross-validation

To further assess the models’ performance, the data were also partitioned randomly in a fivefold cross-validation approach. The predictive ability of the cross-validations differed from when the data training test sets were assigned by cycles of selection, for four of the seven traits (Table  4). The fivefold cross-validation allowed for a more ideal situation when computing prediction modeling. Compared to our partitioning based on cycles of selection, a cross-validation approach increases the genetic overlap between the training and test set, and limits the relative size of the test set (Daetwyler *et al.* 2013). TW, TN, ATW, and SG were traits that showed an increased predictive ability when using the cross-validation approach, compared to our approach of partitioning the training and testing sets. However, accuracy for prediction of traits such as Size, Shape, and Eye was very similar for both partitioning methods.

### Evaluation of predictive power across and within families

GEBVs were predicted using the two largest full-sib families from the first clonal generation, T_1_. Four phenotypic traits were available for the two families, and were predicted using model (2). The prediction model was built using the phenotypic data from one family (T_1_-C or T_1_-D) with the genetic relationship between individuals, and the phenotypic values were estimated for the other family (T_1_-C or T_1_-D). All traits except TN were predictable using the linear model (Table  5). The predictive ability was similar regardless of which of the two families were used as test set and training set. The highest predictive ability was found for LB (0.29–0.31), while TW and ATW had relatively low predictive ability. In addition to the across family prediction, GEBVs were also predicted within the families separately using randomly assigned fivefold cross-validation. The predictive ability was greater for three out of four traits when using cross-validation, compared to when the data was partitioned into training and test sets according to family grouping (Table  5). However, accuracy in prediction of LB was lower when using the cross-validation approach. The predictive ability for the four traits differed depending on which of the two families was used in the model. The greatest difference in predictive ability between the two families was for TN, where predictive ability was 0.07 for cross-validation within T_1_-C and 0.26 for cross-validation within T_1_-D.

**Table 5:** Prediction accuracy (correlation between observed and predicted phenotypes) for four breeding traits: tuber weight per plant (TW), tuber number per plant (TN), average weight per tuber (ATW) and host plant resistance to late blight (LB)

Training population	Test population	Breeding trait			
		TW	TN	ATW	LB
T_1_-C	T_1_-D	0.088	–	0.101	0.29
T_1_-D	T_1_-C	0.069	–	0.080	0.31
T_1_-C^*^		0.130	0.070	0.337	0.25
T_1_-D^*^		0.300	0.260	0.182	0.16

Model validation was conducted on individuals divided by full-sib family or by randomly partitioned fivefold cross validation within each full-sib family. The symbol “-” indicates that the prediction accuracy for the trait was negative, *i.e.*, prediction was not possible.

* For the cross validation within the full-sib family, individuals were divided at random, mean prediction accuracies were estimated over 100 model runs.

## Discussion

As proposed by Bradshaw (2017), introducing genomic selection for the first year of clonal selection would have a large impact on the efficiency of a potato breeding program. At this stage in the potato breeding cycle, the number of replications is the limiting factor for accurate phenotyping. Many key breeding traits, such as tuber yield, shape and size, cannot be accurately assessed until later vegetative generations, where more tubers from the same clone are available for planting in the field. In this study, we attempted to implement the proposition described above by implementing genomic selection in a potato crossbreeding program that includes several different generations of clonal selection.

The degree of population structure differed greatly among the different populations (Figure  1). It was clear that the individuals in the T_1_ generation had very limited genetic overlap with the populations made up of later clonal generations. This was likely because the population T_1_ included a very small number of parents in the pedigrees, while T_2_ and T_3_ had a broader parentage, thus making the effective population size of T_1_ much smaller than those of the later clonal generations. We theorized that the limited genetic overlap and difference in effective population size between T_1_ and the other populations could influence the predictive ability when using T_2_ and T_3__–__7_ as training sets for predicting GEBVs with genomic prediction models. The overall low accuracy of prediction for tuber yield traits, compared to the prediction accuracy estimated in previous research, is most likely due to the relatively large populations used to validate the GEBVs in our experiments (Desta and Ortiz 2014). The genetic overlap among the five full-sib families from T_1_ was remarkably limited, considering that every family shares one crossing parent with at least one other family (Table  2, Figure  1, Supplementary Figure S1). We infer that this clear population structure among the five families may occur due to the nature of tetrasomic inheritance.

Comparing our results to results from previous research on the use of GEBVs for selection in potato breeding is difficult, as methods to estimate trait predictive ability, population structure of the investigated population, and methods of partitioning test and training sets vary greatly. Previous research indicated that tuber yield had a prediction accuracy of above 0.5 (Stich and Van Inghelandt 2018) using cross-validations. However, when distinct sets of training and test populations were used (Habyarimana *et al.* 2017; Endelman *et al.* 2018), the accuracy for tuber yield was estimated as not exceeding 0.3. Previous studies that used division of training-test sets had a maximum of 30% of the total number of individuals as a test set, while in our study, some divisions were of the same proportions, and some were divisions where the test set makes up more than 50% of the total number of individuals. Since the number of individuals is far greater in the early stages of clonal selection compared to the advanced stages in a crossbreeding program, using a training population of a smaller size than the targeted test population could be difficult, but this is a challenge to circumvent. Our prediction accuracies, using the fivefold cross-validation approach, are consistent with those of cross-validation models available in the literature (Habyarimana *et al.* 2017; Stich and Van Inghelandt 2018; Sverrisdóttir *et al.* 2018; Caruana *et al.* 2019); *i.e.*, the estimates for tuber yield are within the 0.3–0.54 range, for dry matter or starch content or SG within the range 0.3–0.8, and for eye depth 0.54.

Studies on the power of GEBVs estimated within and across full-sib families have proven these methods successful for breeding other crops such as, for example, wheat, maize, and sugar beet (Zhao *et al.* 2015). To our knowledge, this study is the first to predict GEBVs across and within full-sib families of potato (Table  5). The prediction accuracies obtained for TW and ATW across the two T_1_ families (T_1_-C and T_1_-D) were similar to the prediction accuracies obtained across different generations of selection (Table  4). LB yielded the highest accuracy out of the traits scored for T_1_, and unfortunately, this trait was not scored for the advanced T_i_ generations. LB has been included in other previous genomic selection studies on potato. The prediction accuracy for LB we obtained in the across family predictions are similar to what Enciso-Rodriguez *et al.* (2018) found using randomly partitioned cross-validations. According to our results, predicting LB across full-sib families gave higher accuracy than within the same family. This is not in line with previous research on genomic selection for biparental families, where full-sibs yield higher prediction accuracies than half-sibs (Brauner *et al.* 2020). TW, TN, and ATW all had higher prediction accuracies for the within family predictions, but the accuracies varied depending on which of the two families the prediction was for. One family did not consistently have higher prediction accuracies than the other, but the results depended on which trait was being predicted. The difference in prediction accuracies between the families might be due to the genetic background of the individuals, or the different spread of phenotypic ranges between T_1_-C and T_1_-D, or the variance among cross-validations (Supplementary Figure S1 and Table S5).

The predictions for TW and TN are low regardless of whether the training-test sets were set up between different generations of selection (Table  4). The exception for this trend was noted for predictions of ATW, where a slightly higher prediction accuracy was observed after partitioning the split between training and test sets. The predictions for ATW were less accurate when using the T_2_ and T_3__–__7_ populations as a training set for GEBVs of the T_1_ population. One reason that ATW shows a higher predictive ability compared to TN and TW could be the distribution of the phenotypic data for these traits (Figure  2, A–C). On average, the adjusted means per location for TW and TN differed greatly; *i.e.*, values were lower for population T_1_ compared to populations T_2_ and T_3_. The values for ATW were much more homogenous over the three populations. Hence, the lower prediction accuracy for TW and TN could be due to the range of phenotypic variation found in each of the two clonal selection generations (unselected T_1_, compared to T_2_ and T_3_ which have undergone at least one cycle of selection) not being represented in the generation used for training the prediction model.

The predictive ability of GEBVs for SG has been estimated in previous research on the potential of genomic selection for potato breeding, either by estimating it as tuber starch content (Sverrisdóttir *et al.* 2017; Stich and Van Inghelandt 2018) or, as here, as the direct measure of the SG or the dry matter content in the tubers (Habyarimana *et al.* 2017; Endelman *et al.* 2018; Sverrisdóttir *et al.* 2018). The prediction accuracy for SG in our research using generation-based partitioning is lower than the prediction accuracy estimated in the previous cited research, but still within their range of estimations depending on the method and partitioning of training and test sets used for the prediction models.

The heritability estimates for our population are, to a very limited degree, reflected in the prediction accuracy of the phenotypic traits. The highest *H*^2^ was found for SG, which also displayed the highest predictive ability (Table  3). However, high heritability was also found for the tuber yield traits, which displayed very low predictive ability when using our partitioning of training and test sets based on cycle of selection. The predictive ability of the fivefold cross-validation approach (Table  4) gives a better reflection of the high *H*^2^ estimates found in the tuber yield traits. There is a correlation between highly heritable traits and high prediction accuracy of GEBVs (Zhang *et al.* 2017). However, there is not a one to one correlation between trait heritability and cross-validation predictive ability. When looking at the results for all traits collectively, a general trend is apparent. Some of the highest *H*^2^ and prediction accuracy were for TW, TN, and ATW, while Size, Shape, and Eye had the lowest for both. The total numbers of individuals used to produce the cross-validation prediction accuracy were much smaller for SG, Size, Shape, and Eye, which might have affected the model’s predictive ability, since a smaller sample size will result in higher standard errors (Altman and Bland 2005). Trait heritability, on the other hand, was not affected by population size because the same individuals were used for this estimate for the seven traits. LB was not included in the heritability estimates as this trait was not measured in the T_3__–__7_ clones used to calculate *H*^2^. LB has previously been reported to have moderate to moderate-high narrow-sense heritability (ranging between 0.31–0.69, Pajerowska-Mukhtar *et al.* 2009; Solano *et al.* 2014; Enciso-Rodriguez *et al.* 2018).

As previously mentioned, yield traits in potato have low to medium narrow-sense heritability (Ortiz and Golmirzaie 2002; Schönhals *et al.* 2017), which includes additive and digenic variation, while the broad sense heritability (*H*^2^) for these traits in our research may be overestimated by including therein the other nonadditive genetic variation (tri- and quadri-genic intra locus and inter-locus interactions or epistasis). One explanation for this can be found when looking at the standard error of the variance due to genotypic effect, which is very high, for TN per plant (Table  3). Among the other known research on genomic selection in potato, only Stich and Van Inghelandt (2018) included an estimation of heritability for tuber yield (0.77), and even if their prediction accuracy for this trait was higher than ours, taking into account previous results it is still reasonable to think that this high heritability value was overestimated.

To the best of our knowledge, this study is the first to include estimations of GEBVs for tuber uniformity traits, which, according to our results, seem to be partially predictable using GEBVs, but with a low accuracy ranging between 0.08 and 0.2, and depending on the sizes and genetic relationships of the test and training sets. Across generational predictions were also successful for these traits and could probably be improved with a larger population size, *e.g.*, by including data from the T_1_ population.

In conclusion, based on our results and the results of previous research as cited above, we remain cautious about proposing genomic selection as a standard breeding method for improving potato following the approach of across selection cycle predictions. The main problems, if this method is to be introduced as a genomics-based selection approach in a small potato breeding enterprise with a finite population size, are likely to be the large number of individuals included in the test set relative to the training set, and the limited genetic overlap between generations based on marker and pedigree research. The limited pedigree-based relationship between clonal generations seen in this study would, however, not reflect the relationships over generations found in other potato breeding programs. Our T_1_ population is not a typical first clonal generation because of the small number of parents involved. Often, a breeding program would use the same parents for several years in the crossing blocks, and this may lead to more genetic overlap between the clonal generations than in those used in our study. However, such an approach may lead to inbreeding, which is undesirable in potato breeding. On the matter of the limited size of the training population relative to the test population, the inclusion of historical data to train the model will be an option once genomic selection has been introduced as a method in the breeding program for a few years. This would increase the genetic diversity and population size of the training set used to train the model to develop GEBVs, and hence increase the predictive ability of the genomic selection model (Isidro *et al.* 2015; Cericola *et al.* 2017; Sverrisdóttir *et al.* 2018). With the data available from this finite size breeding program, we have demonstrated that genomic prediction for selection based on GEBVs would be hard to implement for various target potato breeding traits using cross population predictions. Though the implementation of genomic selection in early clonal generations would limit the number of clonal generations needing to be evaluated using costly and labor-intensive field trails every year, the predictive ability using later cycles of selection to predict the performance of this material is not adequate following this approach. The predictive ability values across full-sib families are also very low for tuber yield traits, though relatively high for LB. The best prediction accuracies were obtained using within family predictions, and following this approach could make potato breeding more efficient as one could phenotype only a portion of the offspring produced in bi-parental crosses. However, the accuracy will vary depending on the genetic background of the trait of interest and the number of full-sibs in the family.

## Data availability

The datasets generated in this study are available through figshare, https://doi.org/10.25387/g3.14376938. The datasets include genotypic and phenotypic data for the 669 potato genotypes, parents, and breeding clones in the SLU potato breeding program.
